# Rhodiola Rosea for Mental and Physical Fatigue in Nursing Students: A Randomized Controlled Trial

**DOI:** 10.1371/journal.pone.0108416

**Published:** 2014-09-30

**Authors:** Salima Punja, Larissa Shamseer, Karin Olson, Sunita Vohra

**Affiliations:** 1 Department of Medicine, Faculty of Medicine and Dentistry, University of Alberta, Edmonton, Alberta, Canada; 2 Clinical Epidemiology Program, Ottawa Hospital Research Institute, Ottawa, Ontario, Canada; 3 Department of Epidemiology and Community Medicine, Faculty of Medicine, University of Ottawa, Ottawa, Ontario, Canada; 4 Faculty of Nursing, University of Alberta, Edmonton, Alberta, Canada; 5 Complementary and Alternative Research and Education (CARE) Program, Department of Pediatrics and School of Public Health, Faculty of Medicine and Dentistry, University of Alberta, Edmonton, Alberta, Canada; The National Institute for Health Innovation, New Zealand

## Abstract

**Background:**

Fatigue is one of many unintended consequences of shift work in the nursing profession. Natural health products (NHPs) for fatigue are becoming an increasingly popular topic of clinical study; one such NHP is *Rhodiola rosea.* A well-designed, rigorously conducted randomized controlled trial is required before therapeutic claims for this product can be made.

**Objective:**

To compare the efficacy of *R. rosea* with placebo for reducing fatigue in nursing students on shift work.

**Design:**

A parallel-group randomized, double-blinded, placebo-controlled trial of 18–55 year old students from the Faculty of Nursing from the University of Alberta, participating in clinical rotations between January 2011 and September 2011.

**Interventions:**

Participants were randomized to take 364 mg of either *R. rosea* or identical placebo at the start of their wakeful period and up to one additional capsule within the following four hours on a daily basis over a 42-day period.

**Outcomes:**

The primary outcome was reduction in fatigue over the 42-day trial period measured using the Vitality-subscale of the RAND-36, cross-validated by the visual analogue scale for fatigue (VAS-F). Secondary outcomes included health-related quality of life, individualized outcomes assessment, and adverse events.

**Results:**

A total of 48 participants were randomized to *R. rosea* (n = 24) or placebo (n = 24). The mean change in scores on the Vitality-subscale was significantly different between the study groups at day 42 in favor of placebo (−17.3 (95% CI −30.6, −3.9), p = 0.011), The mean change in scores on the VAS-F was also significantly difference between study groups at day 42 in favour of placebo (1.9 (95% CI 0.4, 3.5), p = 0.015). Total number of adverse events did not differ between *R. rosea* and placebo groups.

**Conclusion:**

This study indicates that among nursing students on shift work, a 42-day course of *R. Rosea* compared with placebo worsened fatigue; however, the results should be interpreted with caution.

**Trial Registration:**

Clinicaltrials.gov NCT01278992

## Introduction

Current evidence does not delineate a single-source of fatigue in the workplace; however, it has been hypothesized that long work hours, long hours of physical or mental activity, insufficient break time between shifts, inadequate rest, excessive stress, or a combination of these factors may be associated with fatigue. Disrupted circadian rhythms have been found to be associated with changes in mental and physical performance [1], [2]. This is of particular importance for those involved in shift work since some physiological functions are optimally regulated according to circadian rhythms, suggesting that the body may not be well suited for shift work.

In 2011, the Government of Alberta employed approximately 33,000 registered nurses [3], of which almost one-third participate in shift work [4]. Fatigue is experienced by 19–29% of shift workers [5]. It has been suggested that fatigue may contribute to medical errors thereby causing negative health outcomes [6]. A study conducted in 2008 identified 205 clinical errors and adverse incidents (e.g., medication errors, patient falls) reported by 201 night shift nurses over a one year period [7].

There is no specific medical treatment or cure for physical or mental fatigue. Treatment, if used, is aimed to reduce symptoms of fatigue such as loss of memory and concentration, headache, sleep problems, and/or exhaustion. Such treatments may include anti-anxiety medications, antidepressants, and dietary changes. However medications such as antidepressants or stimulant medications may have negative consequences such as dependency and other adverse effects [8].

The use of natural health products (NHPs) for fatigue is becoming an increasingly popular topic of clinical study due to their perceived benefit and low risk of harm [9]. *Rhodiola rosea* is an NHP that has been part of traditional medical systems in Europe, Asia, and Russia for centuries, touted for its purported capability to improve physical and mental performance. Its therapeutic capacity lays in its putative adaptogenic and ergogenic properties. An adaptogen is an NHP which produces a non-specific response, and has a normalizing physiologic effect [10] which is believed to increase one’s resistance to stress and fatigue. Moreover, as an ergogenic aid, we hypothesized that *R. rosea* would enhance physical and mental performance.

Two recent systematic reviews, one of which was conducted by our research team, revealed preliminary evidence that *R. rosea* may enhance physical and mental performance as well as reduce symptoms of fatigue [11], [12]. The authors of both reviews (11 trials, n = 514 participants), agreed that the current research suffered from major methodological flaws ranging from poor outcome measurement (i.e. use of non-validated tools), to small sample sizes ands lack of clarity around whether appropriate bias-reducing mechanisms were in place (i.e. random sequence generation, mechanism of allocation concealment, etc). In addition, both sets of authors found that none of the randomized controlled trials were adequately described (i.e. missing important details about the intervention and outcome), when evaluated using the CONSORT (Consolidated Standards of Reporting Trials) Statement [13]. As such, a well-designed, rigorously conducted and clearly reported randomized controlled trial is needed in order to determine *R. rosea’s* true efficacy on mental and physical fatigue.

Of the many challenges to NHP research, minimizing product heterogeneity and promoting dose standardization, were of paramount concern when selecting a formulation for this study. Unlike drug development where a drug is developed around an active compound with known physiological effects, NHP development works in the reverse order: plants have often been consumed by humans for their potential health benefits, sometimes for hundreds of years, before scientific investigation attempts to identify and isolate its active compound(s). In addition, the therapeutic effects of most NHPs are not typically attributable to a single compound. Instead, a number of compounds, termed active constituents, may be responsible for an NHP’s effect. To date, there is lack of clarity around which specific compounds are considered active constituents in *R. rosea*, however, most preparations are standardized to one or both of two compounds – rosavin and salidroside – which naturally occur at a 3∶1 ratio in the plant. Rosavin is unique to *R. rosea*, while salidroside is common to other Rhodiola species.

The primary objective of this trial is to compare the efficacy of *Rhodiola rosea* with placebo for reducing fatigue in nursing students involved in shift work.

## Methods

A double-blind, placebo-controlled, randomized parallel-group trial was carried out in Edmonton, Canada with the approval of the Health Research Ethics Board at the University of Alberta. All participants provided written informed consent. This trial was registered at clinicaltrials.gov with the identifier: NCT01278992 (http://clinicaltrials.gov/ct2/show/NCT01278992?term=NCT01278992&rank=1). The protocol for this trial and supporting CONSORT checklist are available as supporting information; see Checklist S1 and Protocol S1.

### I. Participant eligibility

Fourth year nursing students aged 18–55 enrolled at the University of Alberta, embarking on their first shift-work rotation (rotating or permanent overnight) and otherwise healthy, were eligible for participation in this trial. Students were excluded if they were: breastfeeding or pregnant women; female participants of child bearing potential not practicing a form of birth control throughout the trial; presence of a primary medical condition associated with fatigue (e.g. cardiac, gastrointestinal, respiratory, renal, rheumatologic, or oncologic disease); presence of any neurological or mental health condition; presence of diabetes; concurrent utilization of hypoglycaemic agents; known allergy or hypersensitivity to *R. Rosea* or Sedum family extracts of pollen; known allergy to microcrystalline cellulose or silicon dioxide; concurrent utilization of a stimulant drug; concurrent utilization of ginseng or other rhodiola products; regular use of any medication with central nervous system effects; and low blood pressure or history of significant dizziness. Female participants were required to undergo blood pregnancy tests to ensure they were not pregnant at the start of the trial.

### II. Intervention and Comparator

The *R. rosea* formulation evaluated in this study was developed by the Rhodiola Rosea Commercialization Project (a project initiated by Alberta Agriculture and Rural Development, a department of the Government of Alberta). The team developed a standardized methodology of testing for specific *R. rosea* compounds (rosavins, rosarin, rosin) and salidrosides. Seeds were collected by researchers from plants identified as *R. rosea* and confirmed by DNA testing. Seedlings were grown at a government research station and distributed with authenticity certificates, to growers who had completed the Good Agriculture and Collection Practices course. Roots grown for a different number of years in different locations in Alberta demonstrated great heterogeneity with total rosavins contents varying from 0.2 = 10% dry weight. In order to overcome this natural variation, four year old roots were extracted and dried to create a powdered extract that was standardized to 2.8% total rosavins. The extract was tested for and found to be below all standards set for heavy metals, pesticides, and microbial contaminants. The extract was mixed with 29% microcrystalline cellulose and 0.5% silicon dioxide and was formulated into the study material and 182 mg of formulation was encapsulated into bovine-based gelatine capsules that disintegrated in less than 20 minutes and demonstrated a shelf-life stability of up to three years based on total rosavins content (personal communication, Dr. Susan Lutz, Senior Development Officer, Functional Foods and Natural Health Products, Alberta Agriculture and Rural Development). Study placebo containing 95% microcrystalline cellulose 0.5% silicon dioxide was encapsulated in the same gelatine capsules and had the same appearance, volume, weight, odour and taste as the *R. rosea* product.

### III. Allocation procedure

Participants were assigned a study number upon enrolment. A randomization sequence was computer-generated using the EPICORE Randomization System, by the study’s data management team, the Epidemiology Coordinating and Research Centre (EPICORE) at the University of Alberta using block sizes of 4. The *R. rosea* and placebo were then packaged in bottles and consecutively numbered for each participant (i.e. study number) according to the randomization sequence. The study nurse, who was not aware of the allocation code, enrolled and dispensed bottles containing study medication to participants. The study pharmacist retained a sealed copy of the allocation code to be broken in the event of an emergency. The study investigator, study coordinator, outcome assessors and participants were blind to intervention assignment.

### IV. Study procedure

At the onset of their first clinical rotation involving shift work, participants were instructed to take two capsules at the start of their wakeful period each day for 42 days. Since fatigue is a subjective symptom, participants were asked to self-determine their need for one additional capsule (i.e. a half dose), to be taken within four hours of the initial dose.

### V. Follow-up

Each participant was contacted by the study coordinator within three days of starting the study medication in order to review study procedures, including outcome assessment. A second telephone call was made between days 14–20 of the study to inquire about potential adverse events and to remind them of completion of the outcome assessment forms. A final phone call to the study participants was made between days 35–41 of the study to inquire about potential adverse events, remind them of outcome assessment form completion, and schedule a follow-up visit at day 70. Throughout the six week (42 day) study, subjects received daily e-mail reminders containing a link to the online data collection forms. At the day 70 mark, participants were sent an e-mail reminder to complete all outcome assessment forms for the final time. Participants returned any unused medication to the study nurse at their final scheduled visit.

### VI. Outcomes

Measurements for all outcomes were collected using an online data collection and management system developed by the Epidemiology Coordinating and Research (EPICORE) Center at the University of Alberta to which each participant was given password-protected access.

Fatigue was chosen as the primary outcome; however, since there is no widely-agreed upon tool for measuring fatigue in healthy populations and given that no previously conducted studies assessing the efficacy of *R. Rosea* used an adequate, well-described and validated measure of fatigue, in this study two measurement tools were used to assess this outcome for which correlation was later assessed. One tool is the Vitality-subscale of the generic health-related quality of life instrument, RAND-36 [14]. While the RAND-36 may have been designed for patients, there is plenty of evidence that supports its use to assess quality of life in both healthy and ill populations [15], [16]. The Vitality-subscale is comprised of 4 items collectively aimed at measuring fatigue/energy. A higher summary score for this set of items indicates better health (e.g. improved fatigue). Participants completed the RAND-36 at baseline, on days 7, 14, 21, 28, 35, 42, and four weeks (day 70) following completion of study medication, in order to assess sustained effect. In order to determine whether the Vitality-subscale was an adequate measure, a 10-point Visual Analogue Scale was also used to measure fatigue (VAS-F) [17]. VAS scales are typically used to gauge subjective characteristics (e.g. pain, fatigue) that are difficult to measure. Subjects indicate their feelings about a characteristic/symptom along a linear continuum, where a lower score indicates less fatigue. The VAS-F scale was thought to be the best mechanism to verify whether scores on the Vitality-subscale were a reliable indication of fatigue. The VAS-F was completed concurrent to the RAND-36 at baseline and every other week thereafter (days 14, 28, 42) as well as on day 70. This was different from the initial protocol (i.e. VAS-F to be administered on days 7, 14, 21, 28, and 42) due to the properties of the tool.

Secondary outcomes assessed were: i) health-related quality of life as measured by the eight subscales of the RAND-36, including physical functioning, role limitations caused by physical health problems, role limitations caused by emotional problems, social functioning, emotional well-being, general health perceptions and vitality. This questionnaire was assessed concurrent with the primary outcome (baseline, days 7, 14, 21, 28, 35 and 42) since the Vitality-subscale is a subset of this questionnaire; ii) improvement of individually-selected outcomes as measured daily by the Measure Yourself Medical Outcomes Profile (MYMOP) [18]. Since fatigue is subjective and its impact highly variable between individuals, this tool was used to measure change in items of importance to the participant, as identified by the participant; and iii) adverse events in the preceding 24 hours, measured daily. The Data and Safety Monitoring Board (DSMB) reviewed all adverse event data and assessed their causality in a blinded fashion.

### VII. Sample size considerations

Since no previous studies of *R. Rosea* used a validated outcome measure for fatigue or presented data in a manner that could be used to calculate sample size, the sample size for this study was calculated using an effect size of 6.5 from a comparable study of ginseng on fatigue (a comparable product from the same therapeutic class) [19]. Using a two sided, two-sample t-test, a sample size of 64 participants was required to achieve 90% power with a type I error rate of 0.05 to detect an absolute mean difference of 6.5 on the RAND-36 Vitality-subscale between the *R. Rosea* and placebo, assuming a standard deviation of 8.0. While we had originally planned for a 40% drop-out rate, which would increase our intended sample size to 90 participants, problems with recruitment required us to aim for a minimum of 64 participants.

No interim analyses were planned or performed and there were no stopping rules set up for this very short trial (i.e. for harm, benefit, or lack of overall benefit of the intervention).

### VIII. Statistical Analysis

The primary analysis followed intention-to-treat principles. All statistical tests were two-sided and tests were evaluated against 0.05 level of significance. Demographic and health risk factors of the study population at baseline were tabulated and compared by group (*R. rosea* or placebo). Continuous variables were presented as means (standard deviations) or medians (inter quartile ranges) and categorical variables as counts (percentages). Two sample t-tests were used for comparison of continuous variables. The primary outcome of interest was the Vitality-subscale of the RAND-36. The mean change in the Vitality-subscale from baseline to day 42 was compared between treatment groups using a random effects model (random intercepts) in which all of the repeated measurements were used in the modeling. Observations were independently conditioned on the subject-specific intercepts, thus covariance was zero. In the model, effects of any possible confounding factors, if present, were adjusted. The vitality score was cross-validated by VAS-F for fatigue. The correlation between the two measures was assessed using Pearson’s correlation coefficient. The changes in the other subscales of RAND-36: physical functioning; role limitations due to physical health; pain; general health; emotional wellbeing; role limitations due to emotional problems; and social functioning were studied and compared between the groups. For each of these scores, a random effects model was fitted. Similarly, daily changes in MYMOP scores: symptoms 1 and 2; activity and well-being were studied.

Rates of adverse events were compared between the two groups using the chi-squared test at baseline, day 21, and day 42. SAS 9.1 (Cary, N.C.) and STATA 12 (College Station, TX) were used for data management and statistical analyses. SPSS (Michigan) or Splus/R was used for supplemental graphs.

### IX. Changes from Protocol

We had planned to use a newly developed tool known as the Adaptive Capacity Index used to measure a subject’s ability to adapt to stressors; however, due technical difficulties with our database, we were unable to include this measure at the time of study.

## Results

### I. Study Participants

**Figure 1:**
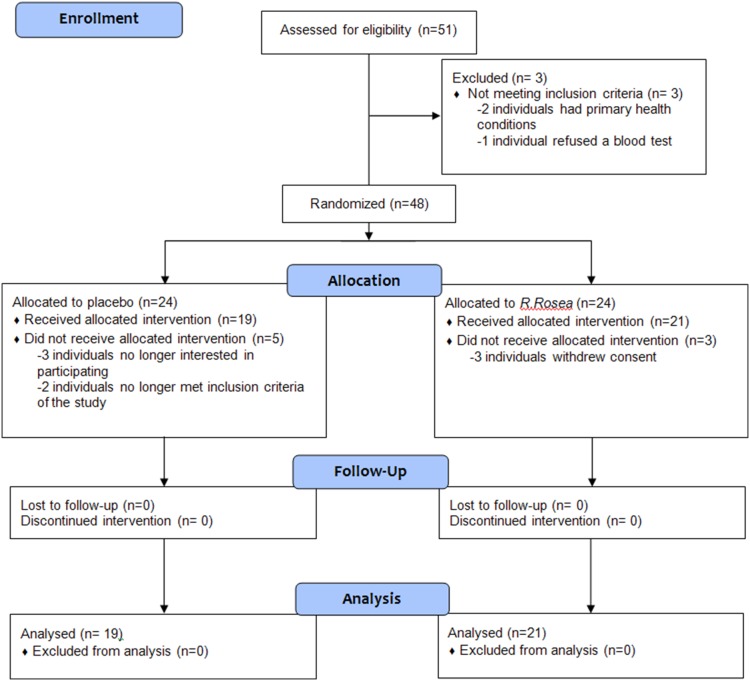
Participant flow.

The intended sample size of 64 participants was not achieved due to a short funding period and subsequent challenges with recruitment. Between January 2011 and September 2011, a total of 51 subjects were assessed for eligibility, of which 48 were suitable and randomized to receive either *R. Rosea* (n = 24) or placebo (n = 24). Six individuals dropped out prior to starting the treatment (three from the placebo arm and three from the *R. Rosea* arm due to no longer wanting to participate), and two participants were withdrawn prior to starting treatment (one participant refused to stop breastfeeding at the start of her trial; one participant was no longer practicing a form of birth control). A total of 40 subjects (n = 21 in *R. rosea* group; n = 19 in placebo group) were included in the analysis. The flow of participants through the trial is described in Figure 1. Baseline demographic data for participants in each group are provided in Table 1.

**Table 1:** Baseline Characteristics of 40 Study Participants.

	Rhodiola (n = 21)	Placebo (n = 19)
Age (yrs); median (IQR)	24 (21–25)	26 (22–29)
Female sex (%)	17 (81.0)	15 (78.9)
Body mass index, median (IQR)^a^	21.7 (21–27)	24.4 (22–27)
Healthy status (%)	21 (100)	18 (94.7)
Smoker (%)	0 (0)	1 (5.3)
On medication prior to randomization (%)	11 (52.4)	7 (36.8)
Married (%)	3 (14)	7 (37)
At least 1 child (%)	1 (5)	1 (5)
Canadian origin (%)	9 (42.9)	11 (57.9)
Aboriginal origin (%)	0 (0.0)	2 (10.5)

Abbreviations: IQR, interquartile range;

a Body mass index is calculated as weight in kilograms divided by height in meters squared.

Although our intended sample size of 64 participants was not reached, a post-hoc power calculation was carried out to compare the mean Vitality-subscale score (of the RAND 36) between placebo and *R. Rosea* taking into account the repeated measures with one at baseline and six follow-up measurements. The correlation between follow-up measurements and baseline were considered 0.7 and 0.4, respectively. At 0.05 level of significance (two-sided), we achieved 81% power to compare mean vitality (SD) of 56.5 (22.7) in the placebo with that of 36.9 (22.0) in *R. Rosea*.

### II. Efficacy of Treatment

The mean change in scores on the RAND-36 Vitality-subscale was significantly different between the study groups (−17.3 (95% CI −30.6, −3.9), p = 0.011), in favor of placebo. Fatigue measured using the VAS-F also significantly improved in favour of placebo (1.9 (95% CI 0.4, 3.5), p = 0.015). Treatment outcomes are presented in Table 2 and Table 3. The rate of change on the VAS-F scale demonstrates similar findings, indicated by an upward trend (worsening of symptoms) in the Rhodiola group; rate of change for the Vitality-subscale is not as clear. A strong inverse correlation between the Vitality-subscale and VAS-F was found for measurements accumulated over the study period confirming that the RAND-36 Vitality-subscale is an appropriate measure of fatigue in this setting (see Table 4).

**Table 2:** Between and within comparison of scores on the Vitality-subscale, RAND-36.

	Rhodiola (n = 21)	Placebo (n = 19)	Mean difference (95% CI)	p-value	
	Mean (SE)	Mean (SE)			
Baseline	49.5 (3.9)	51.8 (4.1)	−2.3 (−13.5, 8.9)		
Day 7	46.9 (4.1)	55.4 (4.4)	−8.5 (−20.3, 3.3)		
Day 14	47.2 (4.5)	55.9 (4.3)	−8.7 (−20.8, 3.4)		
Day 21	45.7 (4.3)	57.4 (4.4)	−11.7 (−23.7, 0.3)		
Day 28	43.2 (4.6)	54.8 (4.6)	−11.6 (−24.4, 1.1)		
Day 35	45.0 (4.4)	54.1 (4.8)	−9.1 (−21.7, 3.5)		
Day 42	36.9 (4.8)	56.5 (5.2)	−19.6 (−33.5, −5.7)	0.006	
**Change from baseline at day 42**					
Mean change (SE)	−12.6 (4.6)	4.7 (5.0)	−17.3 (−30.6, −3.9)		0.011
95% CI of change	(−21.6, −3.6)	(−5.1, 14.5)	-		-

Random effects model was used; Abbreviations: SE. standard error.

Note: Higher vitality score indicates the better vitality.

**Table 3:** Between and within comparison of the VAS-F.

	Rhodiola (n = 21)	Placebo (n = 19)	Mean difference (95% CI)	p-value
	Mean (SE)	Mean (SE)		
Baseline	5 (0.5)	4.4 (0.5)	0.6 (−0.7, 1.9)	
Day 14	5.4 (0.5)	4.5 (0.5)	0.9 (−0.5, 2.4)	0.185
Day 28	5.7 (0.5)	4.2 (0.5)	1.5 (−0.01, 2.9)	0.052
Day 42	6.2 (0.5)	3.7 (0.6)	2.5 (0.9, 4.1)	0.002
**Change from baseline at day 42**				
Mean change (SE)	1.2 (0.5)	−0.7 (0.6)	1.9 (0.4, 3.5)	0.015
95% CI of change	(0.2, 2.3)	(−1.8, 0.4)	-	-

Random effects model was used; Abbreviations: SE. standard error Note: Lower VAS-F score indicates less fatigue (more favorable).

**Table 4:** Correlation between Vitality-subscale and VAS-F.

	Pearson correlation	p-value
Baseline	− 0.741	<0.001
Day 14	− 0.650	<0.001
Day 28	− 0.676	<0.001
Day 42	− 0.899	<0.001

**Figure 2:**
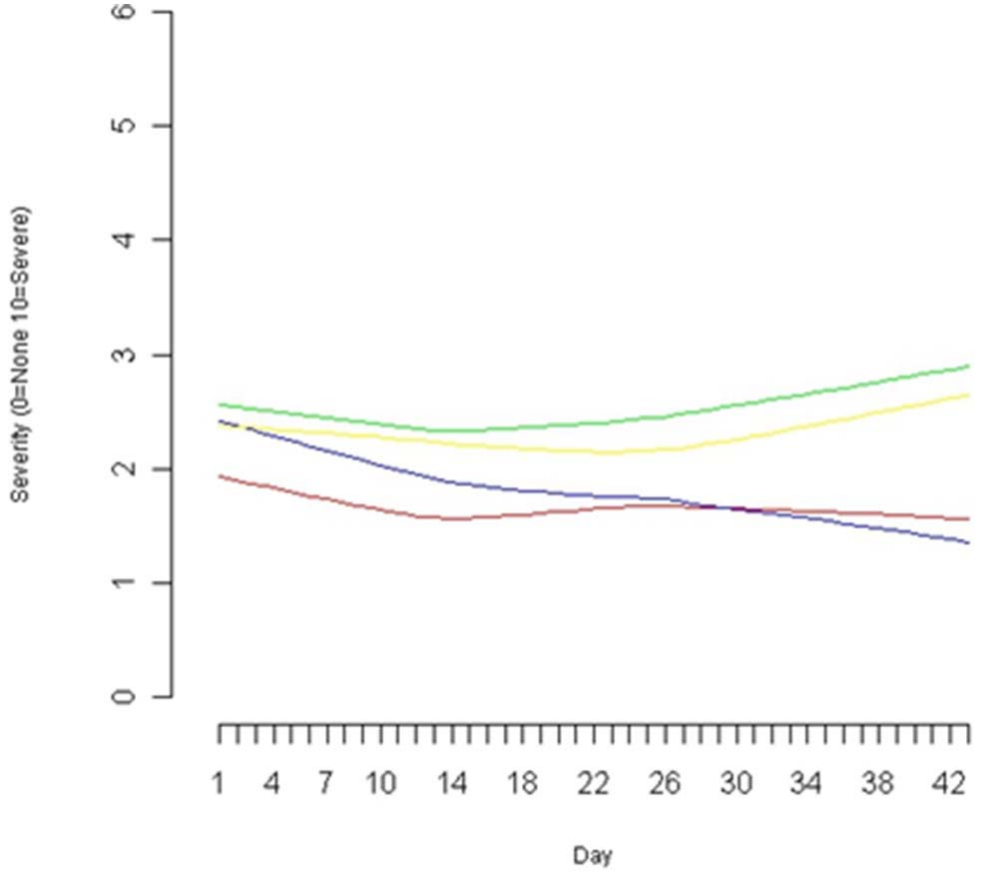
Daily severity of first patient-reported symptom and fatigue on the MYMOP. Legend: Red: First symptom-Placebo Green: First symptom- *R. Rosea* Blue: Fatigue-Placeo Yellow: Fatigue- *R. Rosea.*

The mean difference between change scores for each group of the other seven RAND-36 subscales can be found in Table 5. The only subscale which was significantly different between groups is the general health subscale, in favour of placebo −13.4 (95% CI −24.6, −2.3), p = 0.018. Figure 2 displays the mean change in fatigue symptom scores on the MYMOP, and shows an increase in the score (unfavourable) for the *R. Rosea* group, and a decrease in the mean score (favourable) for the placebo group over time. None of the changes on the MYMOP were statistically significant.

**Table 5:** Between and within comparison of the RAND 36 subscales.

RAND36 Subscale	Rhodiola(n = 21) Mean(SE)	Placebo (n = 19)Mean (SE)	Mean difference(95% CI)	P value
*Physical functioning*				
Baseline	94.5 (1.5)	94.7 (1.6)	−0.2 (−4.4, 4.0)	
Day 42	95.3 (1.9)	96.5 (2.1)	−1.2 (−6.8, 4.5)	
Mean change frombaseline to day 42	0.8 (2.0)	1.8 (2.2)	−1.0 (−6.7, 4.9)	0.754
*Role limitations due to* *physical health*				
Baseline	79.8 (6.3)	88.2 (6.6)	−8.4 (−26.3, 9.5)	
Day 42	63.8 (8.7)	94.4 (9.6)	−30.6 (−55.9, −5.2)	
Mean change frombaseline to day 42	−15.9 (10.7)	6.2 (11.6)	−22.1 (−53.1, 8.7)	0.160
*Pain*				
Baseline	83.8 (4.0)	83.4 (4.2)	0.4 (−11.1, 11.8)	
Day 42	75.5 (5.4)	89.8 (6.0)	−14.3 (−30.2, 1.6)	
Mean change frombaseline to day 42	−8.3 (6.0)	6.4 (6.6)	−14.7 (−32.3, 2.8)	0.100
*General health*				
Baseline	75.5 (3.4)	73.2 (3.5)	2.3 (−7.2, 11.9)	
Day 42	65.0 (4.2)	76.1 (4.6)	−11.1 (−23.3, 1.0)	
Mean change frombaseline to day 42	−10.5 (3.8)	2.9 (4.2)	−13.4 (−24.6, −2.3)	0.018
*Emotional wellbeing*				
Baseline	73.3 (3.5)	74.7 (3.6)	−1.4 (−11.3, 8.5)	
Day 42	66.4 (4.4)	79.6 (4.8)	−13.2 (−25.9, −0.5)	
Mean change frombaseline to day 42	−6.9 (4.1)	4.9 (4.5)	−11.8 (−23.7, 0.1)	0.052
*Role limitations due to* *emotional problems*				
Baseline	65.1 (7.1)	80.7 (7.5)	−15.6 (−35.9, 4.6)	
Day 42	55.0 (8.3)	89.1 (9.0)	−34.1 (−58.0, −10.1)	
Mean change frombaseline to day 42	−10.1 (6.3)	8.4 (7.0)	−18.5 (−37.0, 0.1)	0.051
Social functioning				
Baseline	78.0 (4.5)	81.6 (4.7)	−3.6 (−16.4, 9.2)	
Day 42	71.8 (6.1)	90.2 (6.8)	−18.4 (−36.3, −0.5)	
Mean change frombaseline to day 42	−6.2 (6.9)	8.6 (7.6)	−14.8 (−34.9, 5.2)	0.148

Random effects model was used; Abbreviations: SE, standard error Note: Higher scores indicate better functioning.

### III. Risk of Harm

There were 10 adverse events reported by six participants in the *R. rosea* group and 10 events reported by 6 participants in the placebo group. The specific types and numbers of adverse events are listed in Table 6. All adverse events were categorized as mild to moderate. The DSMB concluded there were no issues of safety of the study products (active or placebo arm). There was no difference in the number or severity of reported harms between study arms.

**Table 6:** Adverse events.

	Rhodiola (n = 21)		Placebo (n = 19)	
Adverse event	Total numberof events	Total number ofParticipantsexperiencing event	Total numberof events	Total number ofparticipants experiencing event
Headache	4	3	2	1
Light headedness	0	0	2	2
Diarrhea/Nausea	2	1	0	0
Dark stool	2	1	0	0
Nausea	1	1	0	0
Nosebleed	0	0	2	1
Blurred vision	1	1	0	0
Excess energy	0	0	1	1
Heartburn	0	0	1	1
Heart palpitations	0	0	1	1
Sore throat	0	0	1	1
**Total**	**10**		**10**	

## Discussion

Our findings indicate that among student nurses partaking in shift work, *R. rosea* worsened symptoms of fatigue, compared to placebo. Significant increases in fatigue in the *R. rosea* group were apparent, while the placebo group did not significantly change over time.

While some evidence suggests that *R. Rosea* may be helpful for symptoms related to physical and mental fatigue, existing clinical studies have methodological flaws which limit accurate assessment of its efficacy. The findings of our study do not appear to be in agreement with previous RCTs in which improvements in fatigue favoured *R. rosea.* However, the major caveat to drawing any comparisons between our study and others are the chosen fatigue measurement tools.

One study examining a similar population to ours – physicians on night duty (n = 56) - evaluated the efficacy of *R. Rosea* against placebo for non-specific fatigue in a cross-over RCT. The authors found a statistically significant improvement on a set of tests, collectively calculated as a ‘Total Fatigue Index’, in favour of the treatment group after two weeks of *R. Rosea* supplementation (p<0.01). The Total Fatigue Index, however, was developed for the purpose of that study and had no history of use or established validity or reliability. In addition, the authors mentioned that physicians were on night duty for ‘considerably’ longer during the second period of crossover than the first, but did not state how much longer or provide rationale. As such, findings of this study must be interpreted as inconclusive. Another RCT compared *R. Rosea* to placebo for improving mental fatigue in 121 male military cadets aged 19–21 years [20]. The investigators reported improvements in favour of assessing *R. Rosea* for fatigue (p<0.001), however, the method of randomization was unclear and similar to the previous study, the outcome measure did not appear to be validated.

Although we designed a rigorous RCT aimed at overcoming the identified shortcomings of previous RCTs, the smaller-than-anticipated sample size achieved left our study vulnerable to potential imbalances in important baseline demographics between the two study groups. We found small imbalances between our treatment and placebo groups in terms of marital status, medication use, and role limitations due to physical and emotional health problems. While these imbalances are likely due to chance, they may account for some of the lack of apparent treatment effect of *R Rosea*. If the participants randomized to *R. Rosea* were less well than those randomized to the placebo group, this would contribute to the lack of perceived effectiveness.

Another possible explanation for our study findings is that the dose used in the study was not sufficient to see an effect. Previous studies of *R. Rosea* for mental and physical performance have reported that in long-term supplementation, up to 680 mg/day is safe and tolerable. Since the participants in our trial had the choice of taking between 2–3 capsules/day, the daily doses ranged from 364–546 mg/day, with most participants taking 364 mg/day (2 capsules). It may be that the average daily dose of *R. Rosea* taken by most trial participants was insufficient to detect beneficial effect.

In a double-blind RCT conducted by Olsson et al, the efficacy of 576 mg/day *R. Rosea* for mental fatigue was assessed in 60 subjects with fatigue syndrome [21]. The authors found significant improvement in favour of *R.rosea* (p = 0.047). Studying patients with more severe fatigue may have helped identify a statistically significant treatment effect that may be less apparent in a healthier population, such as the one we chose to study.

While adverse events were noted in intervention and placebo groups, our study was not powered to detect meaningful differences between the two study arms.

Previous *R. Rosea* research has suffered from the lack of randomization, lack of allocation concealment and blinding, inappropriate outcome measurement tools, inappropriate analytic approaches, and lack of transparency in reporting. Our study overcame these limitations by adhering to rigorous standards for randomization and blinding; items of the CONSORT 2010 checklist were followed in order to ensure transparent reporting [13]. Nevertheless, our results should be interpreted cautiously. We recommend future studies should consider evaluating higher doses of *R. Rosea* with comparable methodological rigor and access to a larger sample in order to determine the effectiveness of *R. rosea*.

## Supporting Information

Checklist S1 **CONSORT Checklist of how trial was designed and analyzed and interpreted.**
(DOC)

Protocol S1 **Study Protocol.**
(DOC)
