# Supplementary material for: Rhodiola Rosea for Mental and Physical Fatigue in Nursing Students: A Randomized Controlled Trial
Source: PLoS One. 2014 Sep 30;9(9):e108416. doi: 10.1371/journal.pone.0108416 (PMC4182456; doi:10.1371/journal.pone.0108416)
Supplement: Protocol S1 — Study Protocol. (DOC) [file pone.0108416.s002.doc]

**A randomized trial of *Rhodiola rosea* for mental and physical fatigue in nurses**

**Protocol Identification #: 2006C002P**

**Trial Sponsor: Alberta Agriculture and Rural Development,** #200 7000 113th Street Edmonton AB, T6H 5T6

Sponsor signatory: **Susan Lutz, PhD,** Sr. Development Officer - Functional Foods and Natural Health Products, Alberta Agriculture and Rural Development

Medical Expert/Principal Investigator/Qualified Physician: Sunita Vohra MD MSc, Professor, Department of Pediatrics, 8B19 11111 Jasper Ave, Edmonton AB, T5K 0L4

**TABLE OF CONTENTS**

[1 BACKGROUND 4](#__RefHeading___Toc275264322)

[1.1 FATIGUE 4](#__RefHeading___Toc275264323)

[1.1.1 CAUSES OF WORK-RELATED FATIGUE 4](#__RefHeading___Toc275264324)

[1.1.2 EFFECT OF FATIGUE ON HEALTH OUTCOMES 4](#__RefHeading___Toc275264325)

[1.1.3 PREVALENCE OF SHIFT WORK AND FATIGUE IN NURSING 4](#__RefHeading___Toc275264326)

[1.1.4 DEFINING FATIGUE 5](#__RefHeading___Toc275264327)

[1.1.5 TREATMENT OF FATIGUE 5](#__RefHeading___Toc275264328)

[1.2 RHODIOLA ROSEA 6](#__RefHeading___Toc275264329)

[1.2.1 IS RHODIOLA ROSEA BENEFICIAL FOR TREATMENT OF FATIGUE? 6](#__RefHeading___Toc275264330)

[1.2.2 IS RHODIOLA ROSEA INDICATED FOR TEATMENT OF FATIGUE FOR NURSES ON SHIFT WORK? 6](#__RefHeading___Toc275264331)

[1.2.3 IS RHODIOLA SAFE FOR USE IN THIS POPULATION? 7](#__RefHeading___Toc275264332)

[1.2.4 RHODIOLA ROSEA FORMULATION TO BE USED 8](#__RefHeading___Toc275264333)

[2 RATIONALE FOR PROPOSED STUDY 8](#__RefHeading___Toc275264334)

[3 OBJECTIVES 9](#__RefHeading___Toc275264335)

[4 Population 9](#__RefHeading___Toc275264336)

[4.1 INCLUSION CRITERIA 9](#__RefHeading___Toc275264337)

[4.2 EXCLUSION CRITERIA 9](#__RefHeading___Toc275264338)

[5 trial design 10](#__RefHeading___Toc275264339)

[5.1.1 RECRUITMENT, informed consent and enrollment 10](#__RefHeading___Toc275264340)

[5.1.2 STUDY VISITS, phone calls and email reminders 11](#__RefHeading___Toc275264341)

[6 trial INTERVENTIONS 11](#__RefHeading___Toc275264342)

[6.1 Treatment regimen 12](#__RefHeading___Toc275264343)

[6.2 DOSE AND DURATION 12](#__RefHeading___Toc275264344)

[6.3 Treatment allocation 12](#__RefHeading___Toc275264345)

[6.4 ALLOCATION CONCEALMENT 13](#__RefHeading___Toc275264346)

[6.5 BLINDING 13](#__RefHeading___Toc275264347)

[6.6 CO-INTERVENTION AND CONTAMINATION 13](#__RefHeading___Toc275264348)

[6.7 ACCOUNTABILITY OF TRIAL MEDICATION 13](#__RefHeading___Toc275264349)

[7 OUTCOME MEASURMENT 14](#__RefHeading___Toc275264350)

[7.1.1 PRIMARY OUTCOME 14](#__RefHeading___Toc275264351)

[7.1.2 SECONDARY OUTCOMES 14](#__RefHeading___Toc275264352)

[7.1.3 WITHDRAWALS/Stopping rules 16](#__RefHeading___Toc275264353)

[8 STATISTICAL CONSIDERATIONS 16](#__RefHeading___Toc275264354)

[8.1 SAMPLE SIZE JUSTIFICATIONS 16](#__RefHeading___Toc275264355)

[8.2 STATISTICAL ANALYSIS 17](#__RefHeading___Toc275264356)

[9 DATA MANAGEMENT 18](#__RefHeading___Toc275264357)

[10 LIMITATIONS 18](#__RefHeading___Toc275264358)

[11 IMPACT OF RESEACH 18](#__RefHeading___Toc275264359)

[Table 1 19](#__RefHeading___Toc275264360)

[Table 2 24](#__RefHeading___Toc275264361)

[Appendix 1: collaborators 25](#__RefHeading___Toc275264362)

[Appendix 2: consort 2010 checklist 26](#__RefHeading___Toc275264363)

[references 28](#__RefHeading___Toc275264364)

1. BACKGROUND

As a profession that necessitates 24 hour access to care, nursing demands a commitment to shift work. Traditionally, shift work involves working shifts outside of the typical daytime hours (1). Typically, permanent night shift (between 11 PM and 7 AM) and rotational shifts (shifts that rotate or change according to a set schedule) are of highest concern in regards to their health effects on nurses (2). It has been shown that the body is exposed to continuous stress when trying to adapt to new working hours (3). The ability to function has been internationally recognized as one of many health implications of nurses on shift work (4).

- 1. FATIGUE
     1. CAUSES OF WORK-RELATED FATIGUE

Current evidence does not delineate a single source of fatigue in the workplace, however, it has been hypothesized that long work hours, long hours of physical or mental activity, insufficient break time between shifts, inadequate rest, excessive stress or a combination of these factors may be associated with fatigue.

Circadian rhythms have been found to be associated with changes in mental and physical performance (5,6). This is of particular importance for those involved in shift work since some physiological functions are optimally regulated according to circadian rhythms suggesting that the body may not be well-suited for shift work.

- - 1. EFFECT OF FATIGUE ON HEALTH OUTCOMES

It has been suggested that fatigue may contribute to medical errors thereby causing negative health outcomes (7). A recent study identified 205 clinical errors and adverse incidents (e.g., medication errors, patient falls) reported by 201 night shift nurses over a one year period (8). A study of hospital-based nurses compared errors in nurses who worked permanent shift compared to rotating shifts and reported twice the odds of reporting a medical error due to tiredness (9). Other studies of hospital-based nurses report similar findings – longer shift hours were found to be associated with increased errors (10).

Some of the reported effects of work-related fatigue that may contribute to such outcomes are: reduced decision-making ability, reduced attention and vigilance, reduced reaction time, increased errors in judgement, increased forgetfulness and increased absenteeism. A large health technology assessment report conducted for the Agency of Healthcare Research and Quality assessed the aspects of fatigue that concerned sleep and their effect on medical errors, states that although fatigue is common cited as a risk factor for medical errors, it may in fact be over-represented in situations that lead to error and must be further studied (11).

- - 1. PREVALENCE OF SHIFT WORK AND FATIGUE IN NURSING

Approximately 20% of the US workforce participates in shift work (12) including almost one-third of full-time nursing professionals (2). Nineteen to 29% of shift workers are thought to experience fatigue (13). Studies comparing shorter shifts (8 hr) to longer shifts (12 hr) found that while some workers preferred the benefits of the longer shift (i.e. shorter work week), fatigue and decreased alertness was experienced near the end of the shift (14). One large cohort study including female nurses found shift work, particularly night duty (*p*<0.001), to be the most important predictor of fatigue (15).

- - 1. DEFINING FATIGUE

Fatigue is a subjective and heterogeneous experience, making an exact definition difficult to capture. Since the proposed study intends to measure work-related fatigue, we will refer to fatigue as that outlined by the Canadian Centre for Occupational Health and Safety: the state of feeling very tired, weary or sleepy resulting from insufficient sleep, prolonged mental or physical work, or extended periods of stress or anxiety (16).

- - 1. TREATMENT OF FATIGUE

Work-related fatigue may be best-managed by better regulation of working hours, especially for those involved in shift work. In Canada and the US, nursing work hours are self-regulated and nurses are expected to use professional judgment to determine whether fatigue might interfere with their performance and, if so, to refrain from practising. Although there is general consensus that a reduction in working hours is necessary, there is little incentive to do so.

A variety of educational and administrative interventions have been employed to help health care personnel adapt to working shifts however, these are difficult to implement on an already strained health care system. There is currently no cure or specific medical treatment for fatigue. Treatment, if used, is aimed to reduce symptoms of fatigue such as loss of memory and concentration, headache, sleep problems and exhaustion. Such treatments may include: anti-anxiety medications, antidepressants, and dietary changes. However, some medications, such as antidepressants or stimulant medications may have unintentional consequences such as dependency (17).

Natural health products (NHPs)[[1]](#footnote-2), such as Echinacea for the common cold, are often explored for health maintenance and treatment of illness because of their perceived benefit and low risk of harm (18). However, there is a shortage of adequate, rigourous, clinical evidence on which to base therapeutic claims. Despite this, North Americans are increasingly using NHPs. In Canada, 70% of people were had used at least one NHPs in 1999 and 26% had used three or more; these number grew to 75% and 31%, respectively, by 2001 (19).

NHPs for stress and fatigue is becoming an increasingly popular topic of clinical study. For instance, a Cochrane systematic review examining Traditional Chinese Medicine for fatigue is currently being conducted (20). Another review of melatonin for sleep disorders, included an examination of the NHP for shift work in which five randomized controlled trials (RCTs) were identified (21,22). The review found that melatonin did not significantly reduce sleep problems (sleep onset latency and sleep efficiency) associated with shift work. Other NHPs have been studied according to their ability to help the body to adapt to different conditions of stress (23-25); one such NHP is *Rhodiola rosea*.

- 1. RHODIOLA ROSEA

*Rhodiola rosea* is a flowering biennial grown at high altitudes in northern latitudes and has been a part of traditional medicine systems in parts of Europe, Asia and Russia for centuries. Due to its adaptogenic properties, it has received attention from the scientific community. The term adaptogen refers to natural herbal products most commonly which are “non-toxic in normal doses, produce a non-specific response and have a normalizing physiologic influence” (26). Similarly, *R. rosea* has been referred to as an ergogenic aid - an herb used to enhance physical and mental performance. Common health indications in *R. rosea* literature pertain to its performance-enhancing and mental stress-reducing properties. Further claims of *R.rosea’s* stress alleviating properties along with immune enhancement and influence on sexuality have recently been showcased in the lay media (e.g. *The Oprah Winfrey Show*). The published literature suggests that these claims may not be well-substantiated.

- - 1. IS RHODIOLA ROSEA BENEFICIAL FOR TREATMENT OF FATIGUE?

A systematic review conducted by our research team identified 11 clinical trials examining the effect of R. rosea and on physical endurance and mental fatigue (Table 1) (27). Two of four RCTs examining *R. rosea* alone for physical performance indicate that the herb, standardized to 3% rosavins, may increase exercise endurance capacity (28,29). Two RCTs examining *R. rosea* in combination with other substances for this indication found that *R.rosea* had no significant effect on physical performance. Of five RCTs of *R. rosea* for enhancing mental performance identified, three indicated that the herb may be effective in improving overall health in a mentally fatigued population (30-32), however in two of these studies, fatigue measurement tools do not appear to be validated, raising concern about the overall validity of their findings. The two remaining studies of mental fatigue offer inconclusive or negative results. Research regarding *R.rosea* efficacy is contradictory. While some evidence suggests that the herb may be helpful for enhancing physical performance and alleviating mental fatigue, the literature reviewed has many methodological flaws and limits accurate assessment of *R. rosea’s* efficacy. A well-designed RCT is needed to determine the true efficacy of *R.rosea* for physical or mental fatigue*.*

- - 1. IS RHODIOLA ROSEA INDICATED FOR TEATMENT OF FATIGUE FOR NURSES ON SHIFT WORK?

Two trials were identified which examine *R. rosea* for fatigue in subjects participating in shift work. One study evaluated *R. rosea* for non-specific fatigue was evaluated in a double-blind crossover RCT in 56 physicians on night duty (33). Participants were randomized to either 170 mg *R. rosea* (standardized to 2.6% salidroside) or placebo. The study period lasted for two weeks followed by a two-week wash-out period, after which participants received the opposite intervention for two weeks. The primary outcome was fatigue, measured using a fatigue index which authors state was specifically developed for use in this study; the tool does not appear to be validated. It is comprised of a composite of scores on five tests that aim to measure speed of visual and auditory perception, attention capacity and short-term memory. Measurements were carried out before and after the treatment period. Authors indicate a significant improvement in the fatigue index after two weeks of *R. rosea* supplementation but provided no statistical evidence for this conclusion. In addition, the authors mention that physicians were on night duty for ‘considerably’ longer during the second period of crossover than the first, however do not state how much longer or provide rationale. As such, findings of this study must be interpreted as inconclusive. Further RCTs with a transparent analytical approach are needed.

Another RCT conducted in Russia evaluated the effect of two different single doses of *R. rosea* on mental fatigue in 121 male military cadets (19-21 years) (31). Subjects were randomized to take 370 mg or 555 mg of *R. rosea (s*tandardized to 2.4% salidroside) or placebo. A non-treatment group was also included, however subjects were not randomized into this group and comparisons against this group is not considered when drawing conclusions. The intervention was taken at 4:00am while participants were on an overnight shift (starting at 6:00pm). Capacity for mental work, measured using a fatigue index of unknown origins, and with no evidence of its psychometric properties, and pulse pressure and rate were evaluated before night duty and one hour after taking the study medication. A self-report questionnaire evaluating general well-being was completed after taking the study medication. The fatigue index was comprised of three parameters: visual perception, short-term memory and perception of order. Improvements in favour of both doses of *R. rosea* were apparent in the fatigue index (*p*<0.001); no significant differences between groups occurred for other outcomes. The method of randomization was unclear and similar to the previous study, the outcome measure does not appear to be validated.

The findings from the above studies are not without major methodological flaws – inappropriate outcome measurement tools and inappropriate analysis approach. The assessment of efficacy of *R.rosea* for fatigue-alleviation in shift-workers would benefit from a well-designed, rigourously conducted RCT.

- - 1. IS RHODIOLA SAFE FOR USE IN THIS POPULATION?

Out of 446 subjects examined in the 11 included clinical studies of physical and mental fatigue, five adverse events were mentioned in three studies. Two subjects on 200 mg of *R. rosea* over a 4-week period each experienced a minor and serious headache (34); one subject on placebo over a 2-day treatment period experienced a minor headache and another had insomnia (29). Another subject on placebo experienced hypersalivation (31). There appear to be few side effects associated with *R. rosea* supplementation; those identified are of a mild nature.

From the available literature, the largest dose of *R.rosea* taken for the longest period of time without side effects is 340 mg twice per day over a six week period. No other adverse effects from case studies or case reports have been identified and available clinical evidence suggests *R. rosea* has a safe profile with little toxicity. The lethal dose of *R. rosea*, at which 50% of animals die (LD_50_), has previously been found to be 3360mg/kg in rats (35). Equivalence calculations have indicated that in adult humans, LD_50_ would be approximately 235,000 mg/kg(36). Clinical studies in the literature report *R. rosea*-only products ranging in dose from 50 mg to 660mg *R. rosea* extract per capsule, to a maximum of 1500mg/day suggesting a large margin of safety. Further, *R. rosea* is available in Canada as a natural health product (i.e. for over-the-counter use as self-care, without a physician’s supervision). In the United States, it is marketed as a dietary supplement which are deemed safe based on a documented history of use or other evidence of safety unless proven otherwise (37). While neither Health Canada nor its US counter-part has issued any safety concerns about *R. rosea,* one product containing *R. rosea* was recalled by the Food and Drug Administration in December 2008 for adulteration with a drug product (hydroxyhomosildenafil – an analog of the active ingredient in a drug used for erectile dysfunction).

Although no contraindications with other herbal or prescription medications currently exist, we postulate that *R. rosea* may have an additive effect with other substances exhibiting stimulant properties and such combination is contraindicated (38). Like many natural health products, the likelihood of adequate reporting of adverse events may be lower than conventional medications due to the inconsistent nature in which patients encounter NHPs – non-standard doses, varying durations and combined with any number of other NHPs or conventional medicines (39).

No safety information currently exists for pediatric, breast-feeding and pregnant populations.

- - 1. RHODIOLA ROSEA FORMULATION TO BE USED

Of the many challenges to NHP research, product heterogeneity and standardization are of paramount concern when selecting a formulation for study. Unlike drug development where a drug is built around an active compound, NHP development works in the reverse order since, in nature, identification of the plant comes before isolation of the compounds it contains. In addition, the therapeutic effects of most NHPs are not typically attributable to a single, compound. Instead, a plethora of compounds, called active constituents, may be responsible for an NHP’s effect.

While it is currently unclear which specific compound(s) in *R. rosea* are active constituents, most preparations of *R.rosea* are standardized to specific levels of marker compounds rosavin, salidroside or both (40). Rosavin is the only constituent unique to the *R. rosea* from the Rhodiola genus; salidroside is common to most other Rhodiola species (41,42). The naturally occurring ratio of rosavins to salidrosides in *R. rosea* is approximately 3:1 and it has been recommended that *R. rosea* preparations reflect this ratio (43).

1. RATIONALE FOR PROPOSED STUDY

Most nurses working in hospital settings are involved in shift work. Although the consequences of this are numerous, many nurses prefer shift work as it allows them flexibility in terms of time and earnings. In addition, it should be noted that in Canada, there are no thresholds or limits to the number of hours worked by nursing professionals. Nursing work hours are self-regulated and development of policies and legislation around this is unlikely forthcoming. As such, some nurses may want an alternative to working fewer hours to help reduce their level of fatigue and/or sleepiness.

From the evidence presented herein, it is apparent that a safe and effective intervention to reduce fatigue in nurses on shift work does not currently exist. Although there have been at least 11 trials examining the effects of *R. rosea* on fatigue or related conditions, none are of adequate quality or methodology to warrant recommendation of use in the target population. Further RCTs of high methodological quality and adequate, evidence-based sample sizes are required to establish the true usefulness of *R. rosea* for fatigue.

1. OBJECTIVES

The primary objective of this trial is to assess whether Rhodiola rosea improves fatigue when compared to placebo in nurses involved in shift work. We will also carry out psychometric testing of the Adaptive Capacity Index.

1. Population

Patients meeting the following inclusion criteria and none of the exclusion criteria will be considered for enrolment in the study.

- 1. INCLUSION CRITERIA

1. Fourth year nursing students enrolled in NURS 495 (Nursing Practice) in the Winter 2011 term at the University of Alberta;
2. Participating in permanent overnight (between 11 P.M. and 7 A.M.) or rotating shift work (shifts that rotate or change according to a set schedule);
3. Otherwise healthy***~~*~~***~~;~~
4. Consent to participate in the study;

***Population justification:*** upon consultation with nurses, nurse managers and the University of Alberta’s Faculty of Nursing undergraduate coordinator, this is the most homogeneous population of nurses and offers a large recruitment base (n>200). Since there is no clear diagnosis criteria for fatigue, all nurses on shift work meeting inclusion criteria will be considered for inclusion.

- 1. EXCLUSION CRITERIA

1. Nurses aged 18 years or younger (safety data for this age group does not exist);
2. Breastfeeding or pregnant women, as confirmed by a blood test (safety data for these groups do not exist);
3. ***Female participants with child bearing potential not practicing a form of birth control throughout the trial;***
4. Presence of a primary medical condition associated with fatigue (e.g. cardiac, gastrointestinal, respiratory, renal, rheumatologic, or oncologic disease);
5. Presence of schizophrenia, bipolar disorder, dementia, eating disorders, insomnia or substance abuse;
6. Presence of diabetes*;*
7. Concurrent utilization of hypoglycaemic agents for raising or lowering blood pressure***;***
8. Known allergy or hypersensitivity to R. rosea or Sedum family extracts or pollen;
9. Known allergy to microcrystalline cellulose or silicon dioxide;
10. Concurrent utilization of stimulant drug such as methylphenidate (Ritalin) amphetamine (Dexedrine, Adderall) methamphetamine (Desoxyn) and pemoline (Cylert);
11. Concurrent utilization of other rhodiola or ginseng products (both fall in the same therapuetic category known as an "adaptogen").
12. Any significant medical condition
13. Any neurological or mental health condition
14. Taking medication that has central nervous system effects;
15. Aged 55 years or older;
16. Low blood pressure or history of significant dizziness
17. trial design

Participants will be randomized to receive either *R. rosea* or identical placebo to be taken daily over a six week period (Error: Reference source not found).

Randomize

Population

Eligible

Sample

Placebo

No intervention

*Rhodiola rosea*

Enrolment (4 weeks before trial start)

Baseline (start of clinical rotation)

Week 10

(End of clinical rotation)

Week 3

Week6

**Figure 1:** Trial Design Schematic

- - 1. RECRUITMENT, informed consent and enrollment

In collaboration with the Faculty of Nursing, students enrolled in NURS 495 will receive notification of the study by way of an introductory email with an information sheet attached in December 2010. The ~~study~~ **project** coordinator will make in-class visits during the first two weeks of January 2011 to answer any study-related questions and to schedule an appointment for eligibility assessment with the study nurse for interested persons. During the eligibility assessment subjects will be given the information sheet to read over and have study procedures explained to them in detail by the study nurse***.*** Subjects will sign the consent form prior to being screened against the selection criteria***.*** All eligible subjects will then be enrolled, randomized and receive their assigned medication from the study nurse during the first visit. However, female participants will need to undergo a blood pregnancy test to ensure they are not pregnant for which they will be given a lab requisition by the study nurse. A total of 2 mL of blood will be withdrawn for this test***.*** When lab results are received by the principal investigator, those with tests confirming they are not pregnant will contacted by the study nurse to confirm their enrolment. Females whose tests indicate positive for pregnancy will be called by the study physician (Dr. Vohra) and asked to return the study medication they received to the study nurse. The participant will then be contacted by the study nurse to schedule a drop-off date and time. During the enrolment visit, the study nurse will also review all outcome measurement tools and walk subjects through a pilot of the tools to be used during the study.

All participants will begin the study on the same, fixed date when their shift work begins (date to be finalized in January 2011). Since data collection will be electronic via the internet, study participants are expected to have internet access. If participants know they will not have internet access or reliable access, arrangements will be made to give them either paper or offline electronic data collection forms (as well as a pre-paid envelope to send the forms back in).

- - 1. STUDY VISITS, phone calls and email reminders

In addition to the initial study visit and phone calls described in section 5.1.1., the project coordinator will phone each subject within 3 days of the study start to review study procedures and outcome assessment documentation procedures. At this time, the project coordinator will inquire about effectiveness of the blind and about participant beliefs about medication effectiveness. The next call from the project coordinator to participants will occur during the 2^nd^ week of the study (days 14 to 20) inquire about adverse effects and to remind them of completion of the outcome assessment forms to be filled out~~.~~ The project coordinator will call study participants during the 5^th^ week of the study (days 35 to 41) to remind them of the forms to be filled out, inquire about adverse effects and schedule a follow-up visit for when they are finished their clinical rotation at the end of 10 weeks. Throughout the study, subjects will also receive daily email reminders containing a link to the online data collection forms for that day. At the 10-week mark (day 70), participants will be sent an email reminder to complete all outcomes assessment forms for the final time. At this time, they will again be queried about their thoughts on effectiveness of the study medication. They will then return any unused study medication to the study nurse during their final scheduled visit.

1. trial INTERVENTIONS

**The following interventions will be supplied:**

1. ***Rhodiola rosea* extract standardized to 2.8% rosavins (**29.0 % microcrystalline cellulose, 0.5% Silicon dioxide
2. **Placebo (**95% microcrystalline cellulose, 0.5% Silicon dioxide)

**Both interventions will be encapsulated in gelatine capsules, which are an animal-based product, and will look identical. Medications will be packaged in identical bottles labelled according to the participants’ randomization code.**

Development of the both interventions will be undertaken by Dr. Raimar Löbenberg, a pharmacist and assistant professor in the Faculty of Pharmacy and Pharmaceutical Sciences at the University of Alberta. Dr. Löbenberg, will store all trial medications (*R. rosea* and placebo) in a secured storage facility until recruitment. Study medication will then be given to the study nurse for dispensing at time of enrolment; medication will be stored under lock and key accessible only by the study nurse until time of dispensing.

- 1. Treatment regimen

The trial will begin on the day that subjects start their clinical rotations and end 6 weeks later. Subjects will be instructed to take two to four capsules of the trial medication per 24 hour period (see section 6.2). All outcomes will be measured at baseline (Day 0) and at the end of the trial period (week 6). The project coordinator will phone participants on the day(s) leading up to their baseline assessment to remind them to complete their questionnaires and ensure they are comfortable with the study procedures. The primary outcome and two secondary outcomes will be measured again at the end of each week; all other outcomes will be measured daily (see section 7). Subjects will be prompted to report adverse events daily during the trial period and at the end of their clinical rotation four weeks after completion of the trial. During the trial period, the study nurse will phone participants in week 2 and week 5 to remind them to complete their questionnaires and to inquire about adverse events (see section 7.1.2).

- 1. DOSE AND DURATION

**Each *R.rosea* capsule will contain 182.0 mg of the product.** Participants will be asked to take two capsules of the trial medication at the start of their wakeful period each day. Since fatigue is a subjective symptom, participants will be asked to self-determine their need for a second dose of the trial medication within the four hour period after the initial dose. The second dose will be one capsule~~;~~ the daily MYMOP will ask participants to record the number of capsules ingested in the past 24 hours.

***Dose & duration justification:*** Since the majority of previous trials that found significant effects assess long-term, rather than acute supplementation of *R. rosea*, this trial will examine efficacy over a similar duration yet with an aim to overcome limitations of previous studies. **Based on dose and duration information previous studies of** R. rosea **for mental and physical performance (Table 2), the maximum dose of** R. rosea **without adverse effects is 1500 mg over three days (short-term supplementation). The maximum dose that has been safely used in long-term supplementation is 680 mg standardized to 3.0% rosavins for a six week period. The *R.rosea* formulation being used in this trial has been standardized to contain 2.8% rosavins; therefore the equivalent maximum daily dose for this study is 546 mg.**

- 1. **Treatment allocation**

**Partcipants will be assigned to a treatment group (either *R.rosea* or placebo) according to a computer generated randomisation scheme produced by our data management team at the Epidemiology Coordinating and Research Centre at the University of Alberta.** Sampling will be stratified by night vs. rotational shift workers and a permuted block randomization technique will be used with permuted block sizes of 4. Although the nursing profession is dominated by females, there has been no consistent association between fatigue and gender and therefore randomization will not be stratified by gender.

**The Investigator will be supplied with individual code-break envelopes for each patient, containing details of the patient’s treatment. Sealed copies of the randomisation code will also be retained by the study pharmacist, Dr. Raimar** Löbenberg**.**

**The code for a patient will only be opened in an emergency, when it is necessary for a patient’s treatment to be disclosed. If a code is broken the investigator will inform the Study Director/Monitor and document the details of the reason in the patient’s case report forms.**

- 1. ALLOCATION CONCEALMENT

Participants will receive a sealed, serially-numbered opaque bottle containing the study medication or placebo depending on their randomised group assignment. They will be provided a six-week supply of their assigned study medication.

- 1. BLINDING

**The placebo capsule will have the same appearance, volume, weight, odour and taste as the *R.rosea* product so that neither the participant nor trial personnel know who is receiving which medication. The effectiveness of the blind will be assessed at the beginning of the study after a single dose via the MYMOP on Day 1. Participants will be asked to guess which medication they think they were allocated to.**

- 1. **CO-INTERVENTION AND CONTAMINATION**

**Since stimulant medications have been identified as potentially hazardous to combine with *R.rosea*, subjects being recruited will be considered ineligible for the trial if they are using such medication. As part of their daily questionnaires, participants will be asked to record any medication used (prescription, over-the-counter, or natural health product) and quantity of caffeine and alcohol consumption in the past 24 hours to ensure that important co-variables can be considered in the final analysis. These questions will be administered as part of the daily MYMOP assessment.**

**From the start of the treatment, participants will be advised not to take any rhodiola or ginseng preparations for the duration of the trial so that only the *R. rosea* treatment will influence trial outcomes. Furthermore, they will be informed that there is a 50% that they may be receiving *R.rosea*, the dose of which is being in this trial is the highest that has been supported by evidence of safety, and cautioned that they would therefore not want to “double-dose” themselves.**

- 1. **ACCOUNTABILITY OF TRIAL MEDICATION**

During the final visit with the study nurse at the end of their clinical rotation, subjects will be asked to return all unused study medication, including all packaging. The study nurse will return all unused medications to Dr. Löbenberg. Dr. Löbenberg will maintain an inventory, which will include details of receipt, dispensing, return and collection of the study medication.

1. OUTCOME MEASURMENT

**Measurements for all outcomes will be collected using an online data collection and management system. Participants who will not have regular internet access during the trial period will be given paper forms to fill out and be asked to return the forms by prepaid mail or in-person at the end of the trial period.**

- - 1. PRIMARY OUTCOME

No clear definition or measurement tool for fatigue exists. The main limitation of fatigue scales is that very few have evidence indicating what constitutes a minimum clinically important difference (MCID) or a reference score for the target population. As such, the vitality subscale of the generic health-related quality of life instrument, RAND-36, will be employed to assess fatigue. The instrument will be administered at baseline, the end of each week (days 7, 14, 21, 28, 35, and 42). Since the vitality scale is not specifically validated for measuring fatigue, a Visual Analogue Scale for Fatigue (VAS-F) (44) will be concurrently administered so it can be compared to the vitality subscale since the VAS-F has proven validity in fatigued and healthy populations (45).

- - 1. SECONDARY OUTCOMES

1. ***Health-related quality of life:*** The RAND-36 is a 36-item questionnaire that assesses eight areas of functioning: physical functioning, role limitations caused by physical health problems, role limitations caused by emotional problems, social functioning, emotional well-being, vitality, and general health perceptions. This questionnaire will be assessed concurrent to the primary outcome (baseline, days 7, 14, 21, 28, 35 and 42) since the vitality subscale is a subset of this questionnaire.
2. ***Individualized outcomes:*** Since fatigue is subjective, and its impact is highly variable between individuals, individualized improvement will be measured using the Measure Yourself Medical Outcomes Profile (MYMOP). This tool will be used to measure change in items of importance to the participant, as identified by the participant. Participants will be asked to complete this short survey daily. Additional questions regarding concurrent medication and sleep hours/quality will be included on the MYMOP. On MYMOP for Day 1, participants will be probed as to their beliefs about which medication they have received in order to test the effectiveness of blinding.
   1. ***Beliefs about efficacy:*** At the beginning of the study, simultaneous to testing the effectiveness of the blind on day 1, participants will be asked how likely they think *R. rosea* is to be effective for fatigue. Participants will be asked the same question at the end of the study.
3. ***Adaptive capacity:*** The Adaptive Capacity Index (ACI) is a 21-item questionnaire that is intended to measure a subjects’ ability to adapt to stressors. The measure has recently been developed and this study aims to provide additional psychometric data about the measure. It is a self-reported measure comprised of five subscales measuring muscle endurance, cognitive function, sleep quality, emotional reactivity and social interaction. It will be administered on the same schedule as the primary outcome.
4. ***Adverse Event Monitoring:*** The daily MYMOP questionnaire has been adapted to query the participant on adverse events within the last 24 hours. A daily report of this item will be sent to the project coordinator once every 24 hours; if adverse events are reported, the study nurse will be notified to follow-up with the participant(s) by phone within 24 hours. For participants completing paper forms, the study nurse will follow-up once per week via phone call to collect information on adverse events. Nurses will record the nature, severity and duration of the adverse event(s). When serious adverse events are reported (i.e. those warranting physician visits or hospitalization), the study nurse will notify the data and safety monitoring board (DSMB). The DSMB will consist of a clinician, a trial methodologist and someone with natural health product expertise who are independent of the trial. In the event of an adverse event, the study nurse together with the qualified investigator may remove a participant from the trial. If the event(s) warrant it, the DSMB is empowered to stop the study prematurely**.** Subjects will also be contacted by phone once every 2 weeks by the study nurse to be queried about adverse event(s) that were not reported. Participants not participating in online electronic data collection will be asked to contact the study nurse within 24 hours of an adverse event occurring. All adverse events will be followed by the study nurse until resolution of baseline status is achieved or until the completion of the trial (i.e. when ethics approval has expired). We will ensure that Health Canada is informed of all serious adverse events within one week of occurrence.

Adverse events are defined as:

- Any untoward medical occurrence in a patient or clinical investigation subject administered a pharmaceutical product and which does not necessarily have to have a causal relationship with this treatment.

Serious adverse events are defined as:

- any untoward medical occurrence that at any dose:
  - results in death
  - is life-threatening, (NOTE: The term "life-threatening" in the definition of "serious" refers to an event in which the patient was at risk of death at the time of the event; it does not refer to an event which hypothetically might have caused death if it were more severe.)
  - requires inpatient hospitalisation or prolongation of existing hospitalisation,
  - results in persistent or significant disability/incapacity
  - is a congenital anomaly/birth defect.

A serious adverse event will result in withdrawal from the trial.

On the third study call, the study nurse will schedule a visit with subjects for the end of their 10-week clinical rotation. During that visit subjects will be asked to return all unused study medication, queried about occurrence of any adverse events after the end of the study period and asked about the effectiveness of study medication.

- - 1. WITHDRAWALS/Stopping rules

Due to the implied high prevalence of fatigue among nurses on shift-work, and anticipated low-risk of side-effects, a high rate of participant withdrawals is not expected. Nonetheless, a 40% drop-out rate has been accommodated in the sample size calculation.

Some participants may choose to withdraw from the study. Reasons for withdrawal will be documented as one of the following:

- Adverse Event (serious or not)
- Death
- Withdrawal of consent
- Protocol violation
- Treatment failure or insufficient response
- Lost to Follow up
- Other

Up to 2 attempts to contact participants who are lost to follow-up will be made by phone or email. Patients who withdraw from the study will be encouraged to undergo all outcomes assessments listed for the final follow-up session.

Those participants, who meet any of the exclusion criteria during the trial, will be withdrawn.

Since there will be no interim analysis performed, there are no stopping rules for the entire trial (i.e. for harm, benefit or lack of overall benefit of the intervention).

1. STATISTICAL CONSIDERATIONS
   1. SAMPLE SIZE JUSTIFICATIONS

Lack of an appropriate, standard, valid tool to measure fatigue has been a barrier to high quality fatigue research. Previous R. rosea has suffered the consequence of this. Since no previous studies of R. rosea used a validated outcome measure or presented data in a manner on which to base sample size, sample size was calculated using the effect size from a comparable study of ginseng on fatigue (a comparable product from the same therapeutic class, i.e. adaptogen) (46). Using a two sided, two-sample t-test, a sample size of 64 participants achieves 90% power to detect an absolute mean difference of 6.5 on the RAND-36 vitality subscale between those randomized to receive R. rosea versus placebo at 0.05 level of significance (two-sided test). Assuming a drop-out rate of 40%, the sample size is increased to 90.

- 1. STATISTICAL ANALYSIS

Analysis will be conducted by the Epidemiology Coordinating and Research (EPICORE) Centre at the University of Alberta.

All primary analyses will apply the intention to treat principles. All statistical tests are two-sided and tests will be evaluated against 0.05 level of significance. Estimates and corresponding 95% confidence intervals will be reported. The RAND-36 vitality subscore will be used as the primary indicator of the efficacy of *R.rosea*. To test the main hypothesis of the effects of *R.rosea* in reducing fatigue of nurses involved in shift work, the mean change from baseline to six weeks of the vitality subscore using RAND-36 will be compared between the *R.rosea* and placebo groups. This 2-sample t-test will be supplemented by additional analyses using rate of change outcome measure. Linear mixed model will be employed to test changes in the intercept and slopes of the treatment groups. This type of modelling accounts for the variability and correlatedness of the observations on the same participant. Despite the tendency of participants to miss testing occasions, the mixed model analyses can still evaluate any trajectories of change. Missing data will be checked for randomness and correlation with adverse events will be explored. Since RAND-36 vitality subscore is not validated for use in this specific population, correlatedness between it and a visual analogue scale for fatigue (VAS-F) will be tested. In addition, correlatedness between ACI, will be tested against the primary outcomes in order to establish further evidence of validity. RAND-36 physical and mental component scores will be analysed by testing their mean change from baseline to day 42 between treatment groups, as a secondary outcome. Mixed model analyses will be applied for testing change for VAS-F, RAND-36, ACI and MYMOP. Side by side graphs of the rates of change between treatment groups will be plotted at the exploratory and final stages of analysis. Examination of the effect of the covariates - concurrent medication, dose-related change, sleep, alcohol use and impact of specific rotation (rotating vs permanent shift-work), age and marital status will be analyzed using linear mixed regression models as secondary outcomes. SAS 9.1 (Cary, N.C.) will be used for data management and statistical models. SPSS (Michigan) or Splus/ R will be used for supplemental graphs.

Adverse events will be monitored daily. Rates of adverse events will be compared between the two groups using the chi squared test at baseline, day 21 and day 42 measurements.

1. DATA MANAGEMENT

All questionnaires will be self-reported and administered through a secured (password-protected) electronic data collection system. Participants will access the online data collection system using their assigned ID (i.e. randomization number) and a randomly generated password. The daily MYMOP questionnaire will be automatically queried daily for adverse event data; this information will be forwarded to the project coordinator once every 24 hours for review. All data will be collected electronically and transferred into statistical software at the end of data collection for cleaning and analysis by a statistician.

1. LIMITATIONS

The generalizability of this trial is limited to nurses in a shift work setting and may not be generalizable to other work settings or the general healthy population. However, as nurses are such a fundamental part of health care, the implications on this population alone are thought to be sufficient to warrant study.

In addition, since no valid approach for defining and measuring fatigue exists, this is a major downfall of previous and future studies. As such, we have decided to measure health related quality of life, since some argue this is fundamentally more important to health than measurement of a particular disease state.

1. IMPACT OF RESEACH

## The proposed trial will provide evidence of efficacy and safety for *R. rosea* in nursing students who are at risk for fatigue due to shift work. If effective, *R. rosea* may be a treatment option for nurses to reduce their levels of fatigue during shifts. This is important since fatigue among health professionals has been shown to impact patient safety medical errors.

Table 1

Summary of clinical evidence for *R. rosea* for indication of mental and physical fatigue (RR: *Rhodiola rosea*; DB: double-blind; RCT: Randomized controlled trial; CCT: Controlled clinical trial; CO:cross-over; SHR-5: Swedish Herbal Institute RR preparation)

| **MENTAL STRESS/FATIGUE** | | | | | | | | | | | | | | |
| --- | --- | --- | --- | --- | --- | --- | --- | --- | --- | --- | --- | --- | --- | --- |
| **Study** | | **Design** | | **N** | | **Population** | | **treatment/control** | | **Treatment regimen** | | **Primary outcome (tool)** | | **Results** |
| Olsson 2009(30) | | DB RCT | | 60 | | Subjects with fatigue syndrome | | 576 mg of *R.* rosea (2.7% salidroside)/ placebo | | Once/day for 28 days | | Mental fatigue (Pines burnout scale) | | Significantly improved in favour of RR (p=0.047) |
| Shevtsov 2003(31) | | DB RCT | | 121 | | Male military cadets on night shift;  19-21 y | | 370 mg RR/555 mg RR/placebo/nothing | | Single-dose taken in middle of night shift; | | Mental fatigue (total anti-fatigue index) | | Significantly improved in favour of RR (p<0.01) |
| Darbinyan 2000(33) | | DB CO RCT; | | 56 | | Physicians on night duty; 24-35 y | | 170 mg RR (2.6% salidroside)/placebo | | 14 day treatment periods with 14 day wash-out period | | Mental fatigue (total fatigue index) | | Data not presented |
| Spasov 2000a(32,47) | | DB RCT (pilot study) | | 40 | | Foreign male students; 17-18 y | | 100mg RR (SHR-5)/placebo | | Daily for 20 days | | Physical work capacity; pulse rate; psychomotor function (maze test, tapping test); mental capacity; general well-being and mental fatigue | | pulse rate (p<0.05), maze test (p<0.01), general well-being (p<0.05) and mental fatigue (p<0.01) significantly improved in favour RR |
| Spasov 2000b (47) | | RCT | | 60 | | Foreign male students; 17-18 y | | 660 mg RR-containing preparation (Rhodaxon)/placebo | | Daily for 20 days | | Physical fitness test PWC-170; pulse rate; mental capacity; neuromotoric fitness; general well-being and self-evaluation questionnaire | | Did not perform between-group comparison |
| **PHYSICAL PERFORMANCE** | | | | | | | | | | | | | | |
| **Study** | | **Design** | | **N** | | **Population** | | **treatment/control** | | **Treatment regimen** | | **Primary outcome (tool)** | | **Results** |
| Walker 2007 (48) | | DB CO RCT | | 12 | | Resistance-trained males; 19-39 y | | RR vs. placebo | | 1500mg daily for 3 days followed by  1000 mg on fourth day; 7-14 day washout before next treatment period | | ATP turnover (nuclear magnetic resonance spectroscopy); | | non-significant difference between treatments |
| Colson 2005 (49) | | DB RCT | | 8 | | Healthy males; 18-34 y | | Herbal preparation containing RR (3% rosavins, 2.5% salidrosides)/placebo; every 3 capsules contained 1000mg CS, 300mg RR and 800mg proprietary blend | | 6 capsules/day for 6 days followed by  3 capsules/day for 7 days | | peak aerobic capacity (peak power, HR, O_2_ and CO_2_ output), physiological reactions (StO_2_ %) and time to exhaustion | | non-significant difference between treatments |
| Earnest 2004 (50) | | DB RCT | | 17 | | competitive male cyclists; 31.1 y | | Herbal preparation containing RR (3% rosavins, 2.5% salidrosides)/placebo; every 3 capsules contained 1000mg CS, 300mg RR and 800mg proprietary blend | | 2000 mg/day for 4 days followed by 1000mg/day for 11 days | | peak aerobic capacity (peak power, HR, O_2_ and CO_2_ output), physiological reactions (StO_2_ %) and time to exhaustion | | non-significant difference between treatments |
| Abidov 2004 (28) | | DB RCT | | 36 | | Healthy adults; 21-24 y | | 340 mg RR/placebo/nothing | | Once daily for 30 days prior to and 6 days following exhausting physical exercise | | C-reactive protein (CRP) and creatinine kinase (CK) levels measured at 5hours and 5 days after exercise | | CRP significantly improved in favour of RR (p<0.05); non-significant difference for CK |
| De Bock 2004a(29) | | DB CO RCT | | 24 | | Physically active students; 18-50 y | | 100 mg RR/placebo | | 2 day treatment period with 5 day washout between periods | | time to exhaustion, peak O_2_ uptake and CO_2_ output, muscle strength, speed of limb movement, reaction time, sustained attention | | time to exhaustion, O_2_ uptake and CO_2_ output significantly improved in favour of RR (*p*<0.05) |
| De Bock 2004b(34) | | DB CCT | | 12 | | Physically active students; 18 – 50 y | | 200 mg RR/placebo | | Daily for 28 days | | time to exhaustion, peak O_2_ uptake and CO_2_ output, muscle strength, speed of limb movement, reaction time, sustained attention | | Non-significant difference between treatments |
| **MENTAL ILLNESS** | | | | | | | | | | | | | | |
| **Study** | **Design** | | **N** | | **Population** | | **treatment/control** | | **Treatment regimen** | | **Primary outcome (tool)** | | **Results** | |
| Bystritsky 2008(51) | Open-label pilot study | | 10 | | generalized anxiety disorder | | 340 mg RR/no control | | Twice daily for 10 weeks | | Symptom improvement (Hamilton Anxiety Rating Scale [HARS] and Clinical Global Impressions Scales for Improvement [CGI-I]) | | HARS (*p*=0.01) significantly improved in favour of RR; CGI-I data not presented | |
| Fintelmann 2007(52) | CCT | | 120 | | subjects with impaired physical cognitive functions; 50-89 y | | RR-containing supplement; 2 capsules at breakfast/one capsule at breakfast and one at lunch; dose not stated | | 12 weeks | | Symptom improvement measured on a 4-point scale (measurement tool not reported); digit connection test; Physician and patient-reported efficacy and safety | | Did not perform between-group comparison | |
| Darbinyan  2007 (53) | DB RCT | | 91 | | Mild/moderate depression; 18-70 y | | 340mg RR/680mg RR/ placebo | | Daily for 42 days | | Hamilton Rating Scale for Depression [HAMD] and [BDI] scores between both RR groups and placebo after treatment. | | Both doses of *R.rosea* significantly improved HAMD and BDI scores over placebo (*p*<0.0001 for both) | |
| **OTHER CONDITIONS** | | | | | | | | | | | | | | |
| **Study** | **Design** | | **N** | | **Population** | | **treatment/control** | | **Treatment regimen** | | **Primary outcome (tool)** | | **Results** | |
| Dieamant 2008a (54) | DB RCT | | 124 | | Subjects with sensitive skin | | serum containing RR (2.8%-3.5% rosavin) and L- carosine associated compound (RCAC)/placebo; dose not specified | | applied to face twice daily for 28 days | | Sting intensity, skin comfort, skin dryness | | Inappropriate analysis performed | |
| Dieamant 2008b(54) | Single arm cohort study | | 39 | | Subjects with sensitive skin | | RCAC/placebo | | Each treatment was applied to opposite arms for 56 days | | Transepidermal water loss | | Did not perform between-group comparison | |
| Ross 2008(55) | Retrospective cohort | | 65 | | Subjects with an eating disorder; 28.6 y | | Integrative medicine program including sleep hygiene, RR-containing supplement and probiotics/standard care | | 6 months | | Difference in self-report and medication use for Insomnia and constipation | | Did not perform appropriate comparison | |
| Meng 2007(56) | RCT | | 118 | | Subjects with Chronic Cerebral Circulatory Insufficiency; 64±3.4 y | | 1000 mg RR-containing supplement/20 mg nimodipine | | Twice daily/thrice daily for 4 weeks | | Treatment effectiveness according to Chinese Health Ministry rating scale | | Did not present comparative analysis | |
| Kormosh 2006(57) | Cohort study | | 28 | | Women with stage III-IV epithelial ovarian cancer undergoing chemotherapy; age not stated | | 270mg of RR-containing supplement (RR*, L.carthamoides, E.senticosus and S.chinensis/*standard care | | Daily for 4 weeks | | t-cells and immunoglobulin levels | | Analysis not presented.  Side effects of chemotherapy (fatigue and depression) noticeably absent in Tx group | |
| Narimanian 2005(58) | DB RCT | | 60 | | acute non-specific pneumonia taking antibiotics; 36.5 y | | 20 ml of RR-containing supplement (27.6% RR, 51.0% *S.chinensis*, 24.4% *E.senticosus*)/ placebo | | Twice daily for 10-15 days | | duration of antibiotic therapy | | Significantly less days on antibiotics in favour of RR-containing supplement (p<0.0001, (5.67 days vs. 7.53 days) | |
| Wing 2003(59) | DB RCT (cross-over between 2 treatments) | | 15 | | Healthy subjects; 25.1 ± 3.7 y | | stabilized oxygen/1778 mg RR/placebo  saline sol’n prior to 30 mins into 1hr hypoxic exposure[[2]](#footnote-3); 8 days later, RR capsules (4capsulesx447 mg/capsule)/day for 7 days; Con: same protocol as above using stabilized O_2_ supplement dissolved in H_2_0 followed by placebo. | | At the beginning of the study period, subjects were given either placebo solution or stabilized oxygen prior to being exposed to hypoxic conditions; 7 days after exposure, subjects on placebo took RR daily for 7 days while those on oxygen were given placebo; | | Blood oxygen level and blood pressure | | No significant difference in blood oxygen levels and blood pressure between groups | |
| Karpova 1999(60) | Single-arm cohort study | | 10 | | Elderly males; 60-74 y | | 45 mL of 40-plant mixture containing RR (no proportion, concentration of constituents) | | Daily dose for 1 month | | Non-specific haematological parameters including number of leukocytes, erythrocytes haemoglobin | | Significant improvement in 6/9 parameters | |
| Bocharova 1995(61) | Single-arm cohort study | | 12 | | Bladder cancer | | 3 mL liquid extract of RR | | Twice daily for 2 months followed by a 2 week wash-out period; this cycle for a total of 18 months | | frequency of relapse, tissue disintegration, immune status | | No significant effect | |

Table 2

Dose and duration of *R. rosea* treatment in physical and mental health studies; *n.s.: not stated; wo: washout*

| STUDY | DOSE (RR extract) | % rosavins | FREQUENCY | DAILY DOSE RR | DURATION (Days) |
| --- | --- | --- | --- | --- | --- |
| Darbinyan 2000 | 170 mg | n.s. | Once/day | 170 mg | 14  14 d wo |
| Shevstov 2003 | 2x185mg  3x185mg | n.s. | single dose  single dose | 370 mg  555 mg | 1 |
| Spasov 2000a | 2x50 mg | n.s. | Not stated | 100 mg | 20 |
| Spasov 2000b | 660mg | n.s. | Not stated | 660 mg | 20 |
| Abidov 2004 | 340 mg | n.s. | Twice/day | 680 mg | 36 |
| Colson 2005 | 2x300 mg  1x300 mg  (herbal supplement) | 3.0% | Once/day  Once/day | 600 mg  300 mg | 6  7 |
| De Bock 2004a | 2x100 | 3.0% | Once/day | 200 mg | 2 |
| De Bock 2004b | 100 | 3.0% | Twice/day | 200 mg | 28 |
| Earnest 2004 | 2x300 mg  1x300 mg  (herbal supplement) | 3.0% | Once/day  Once/day | 600 mg  300 mg | 4  11 |
| Walker 2007 | 2x250 mg  4x250 mg | 3.0% | Thrice/day  Single dose | 1500 mg  1000 mg | 3  1  7 d wo |
| Fintelmann 2007 | 2 capsules  2 capsules  (No dose given) | n.s. | Once/day  Twice/day | N/A | 84 |
| Darbinyan 2007 | 2x170 mg  2x170 mg | 3.0% | Once  Twice/day | 340 mg  680 mg | 42 |
| Bystritsky 2008 | 1x170 mg | n.s. | Twice daily | 340 mg | 70 |

Appendix 1: collaborators

**University of Alberta Collaborators**

| **Role** | **Name** | **Affiliation** |
| --- | --- | --- |
| Principal investigator | Sunita Vohra | CARE[[3]](#footnote-4) Program |
| Co-Investigator | Karïn Olson | Faculty of Nursing |
| Pharmacist | Raimar Löbenberg | Faculty of Pharmacy and Pharmaceutical Sciences |
| Administrator | Linda Majcher | CARE Program |
| Project coordinator | Larissa Shamseer | CARE Program |
| Research assistant | Salima Punja | CARE Program |
| Research nurse | n/a | WCHRI[[4]](#footnote-5) |
| Data management | Lily Yushko | EPICORE[[5]](#footnote-6) |
| Data analysis | Marilou Hervas-Malo | EPICORE |

Appendix 2: consort 2010 checklist

| Section/Topic | Item # | Checklist item | Reported on page # |
| --- | --- | --- | --- |
| Title and abstract | | | |
|  | 1a | Identification as a randomised trial in the title | 1 |
|  | 1b | Structured summary of trial design, methods, results, and conclusions (for specific guidance see CONSORT for abstracts) | N/A |
| Introduction | | | |
| Background and objectives | 2a | Scientific background and explanation of rationale | 4-9 |
|  | 2b | Specific objectives or hypotheses | 9 |
| Methods | | | |
| Trial design | 3a | Description of trial design (such as parallel, factorial) including allocation ratio | 10-11 |
|  | 3b | Important changes to methods after trial commencement (such as eligibility criteria), with reasons | N/A |
| Participants | 4a | Eligibility criteria for participants | 9-10 |
|  | 4b | Settings and locations where the data were collected | 10-11 |
| Interventions | 5 | The interventions for each group with sufficient details to allow replication, including how and when they were actually administered | 11-12 |
| Outcomes | 6a | Completely defined pre-specified primary and secondary outcome measures, including how and when they were assessed | 13-15 |
|  | 6b | Any changes to trial outcomes after the trial commenced, with reasons | N/A |
| Sample size | 7a | How sample size was determined | 15 |
|  | 7b | When applicable, explanation of any interim analyses and stopping guidelines | 15 |
| Randomisation: |  |  |  |
| Sequence generation | 8a | Method used to generate the random allocation sequence | 12 |
|  | 8b | Type of randomisation; details of any restriction (such as blocking and block size) | 12 |
| Allocation concealment mechanism | 9 | Mechanism used to implement the random allocation sequence (such as sequentially numbered containers), describing any steps taken to conceal the sequence until interventions were assigned | 12 |
| Implementation | 10 | Who generated the random allocation sequence, who enrolled participants, and who assigned participants to interventions | 10,12 |
| Blinding | 11a | If done, who was blinded after assignment to interventions (for example, participants, care providers, those assessing outcomes) and how | 12 |
|  | 11b | If relevant, description of the similarity of interventions | 11 |
| Statistical methods | 12a | Statistical methods used to compare groups for primary and secondary outcomes | 16 |
|  | 12b | Methods for additional analyses, such as subgroup analyses and adjusted analyses | 16 |
| Results | | | |
| Participant flow (a diagram is strongly recommended) | 13a | For each group, the numbers of participants who were randomly assigned, received intended treatment, and were analysed for the primary outcome | N/A |
|  | 13b | For each group, losses and exclusions after randomisation, together with reasons | N/A |
| Recruitment | 14a | Dates defining the periods of recruitment and follow-up | N/A |
|  | 14b | Why the trial ended or was stopped | N/A |
| Baseline data | 15 | A table showing baseline demographic and clinical characteristics for each group | N/A |
| Numbers analysed | 16 | For each group, number of participants (denominator) included in each analysis and whether the analysis was by original assigned groups | N/A |
| Outcomes and estimation | 17a | For each primary and secondary outcome, results for each group, and the estimated effect size and its precision (such as 95% confidence interval) | N/A |
|  | 17b | For binary outcomes, presentation of both absolute and relative effect sizes is recommended | N/A |
| Ancillary analyses | 18 | Results of any other analyses performed, including subgroup analyses and adjusted analyses, distinguishing pre-specified from exploratory | N/A |
| Harms | 19 | All important harms or unintended effects in each group (for specific guidance see CONSORT for harms) | N/A |
| Discussion | | | |
| Limitations | 20 | Trial limitations, addressing sources of potential bias, imprecision, and, if relevant, multiplicity of analyses | N/A |
| Generalisability | 21 | Generalisability (external validity, applicability) of the trial findings | N/A |
| Interpretation | 22 | Interpretation consistent with results, balancing benefits and harms, and considering other relevant evidence | N/A |
| Other information | | |  |
| Registration | 23 | Registration number and name of trial registry | N/A |
| Protocol | 24 | Where the full trial protocol can be accessed, if available | N/A |
| Funding | 25 | Sources of funding and other support (such as supply of drugs), role of funders | N/A |

references

1. Defined in Canada as: vitamins, minerals, herbal remedies, homeopathic remedies, traditional medicines, probiotics and other products like amino acids and essential fatty acids that are manufactured, sold, or represented for use in the diagnosis, treatment or prevention of a disease or disorder, for restoring or correcting organic functions or for modifying organic functions in a manner that maintains and/or promotes health. [↑](#footnote-ref-2)
2. 13.6% O_2_ balanced N through hood at barometric pressure of 633 mmHg to simulate 4600m above sea. [↑](#footnote-ref-3)
3. Complementary and Alternative Research and Education Program [↑](#footnote-ref-4)
4. Women and Children’s Health Research Institute [↑](#footnote-ref-5)
5. Epidemiology Coordinating and Research Centre [↑](#footnote-ref-6)
